# Haystack, a web-based tool for metabolomics research

**DOI:** 10.1186/1471-2105-15-S11-S12

**Published:** 2014-10-21

**Authors:** Stephen C Grace, Stephen Embry, Heng Luo

**Affiliations:** 1 Biology Department, University of Arkansas at Little Rock, 2801 South University Ave., Little Rock, AR 72204, USA; 2 University of Arkansas at Little Rock/University of Arkansas for Medical Sciences Bioinformatics Graduate Program, Little Rock, AR 72204, USA

## Abstract

**Background:**

Liquid chromatography coupled to mass spectrometry (LCMS) has become a widely used technique in metabolomics research for differential profiling, the broad screening of biomolecular constituents across multiple samples to diagnose phenotypic differences and elucidate relevant features. However, a significant limitation in LCMS-based metabolomics is the high-throughput data processing required for robust statistical analysis and data modeling for large numbers of samples with hundreds of unique chemical species.

**Results:**

To address this problem, we developed Haystack, a web-based tool designed to visualize, parse, filter, and extract significant features from LCMS datasets rapidly and efficiently. Haystack runs in a browser environment with an intuitive graphical user interface that provides both display and data processing options. Total ion chromatograms (TICs) and base peak chromatograms (BPCs) are automatically displayed, along with time-resolved mass spectra and extracted ion chromatograms (EICs) over any mass range. Output files in the common .csv format can be saved for further statistical analysis or customized graphing. Haystack's core function is a flexible binning procedure that converts the mass dimension of the chromatogram into a set of interval variables that can uniquely identify a sample. Binned mass data can be analyzed by exploratory methods such as principal component analysis (PCA) to model class assignment and identify discriminatory features. The validity of this approach is demonstrated by comparison of a dataset from plants grown at two light conditions with manual and automated peak detection methods. Haystack successfully predicted class assignment based on PCA and cluster analysis, and identified discriminatory features based on analysis of EICs of significant bins.

**Conclusion:**

Haystack, a new online tool for rapid processing and analysis of LCMS-based metabolomics data is described. It offers users a range of data visualization options and supports non-biased differential profiling studies through a unique and flexible binning function that provides an alternative to conventional peak deconvolution analysis methods.

## Introduction

Untargeted metabolomics has become an increasingly powerful tool to investigate biological systems [1-3]. This approach typically employs gas or liquid chromatography combined with mass spectrometry or nuclear magnetic resonance to survey the metabolome and identify features associated with the genotype and/or biological state of the organism [4,5]. Multivariate statistical analysis is then used to model classes and identify important metabolites [6,7].

Similar to other omics disciplines, untargeted metabolomics requires analysis of large multidimensional datasets containing many independent variables [8]. Liquid chromatography-mass spectrometry (LCMS) is the most common analytical platform for these types of studies since it provides the highest sensitivity and broadest coverage of the metabolome [9,10]. LCMS data contain a wealth of information about a sample since metabolites can be diagnosed by both retention time and mass over charge (*m/z*) properties. However, mining LCMS data for important features represents a major bottleneck in metabolomics research. Metabolites that could identify and classify different phenotypes or conditions can easily be missed if they occur in relatively low abundance. Given the enormous range and variability associated with metabolomic data, identifying a distinctive but weak signal from the data pool presents a formidable challenge [11]. Manual identification of these metabolites can be a cumbersome and error-prone task when dealing with large metabolomic studies that involve multiple files and groups.

Efficient and streamlined metabolomics experiments require that data processing be automated with computational tools. A number of server and software applications exist for analysis of metabolomic LCMS data that can help with data pre-processing, visualization, feature detection, and statistical analysis [12-18]. However, there is still a need for flexible web-based custom data visualization and processing tools for researchers in the life and health sciences.

In this paper we introduce Haystack, a versatile web-based tool for processing and analysis of LCMS-based metabolomics data. Haystack features traditional LCMS data processing options along with a unique and fast bin analysis for group classification and possible biomarker identification. It has an intuitive graphical interface and does not require technical expertise in command line programming languages such as R. Raw data files can be uploaded in one of several common formats, including mzData, mzML, mzXML and NetCDF through Haystack's browser interface [10,19]. Haystack can perform a variety of tasks on full-scan LCMS data, including automatic display of the total ion chromatogram (TIC) and base peak chromatogram (BPC), and user-specified display of time-resolved mass spectra and extracted ion chromatograms (EICs) over any mass range. Haystack produces output files of every process in the common .csv format that can be used for further statistical analysis or customized graphing using numerous software/server applications.

The core function of Haystack is a flexible binning procedure that converts the mass dimension of the chromatogram into a set of interval variables that can uniquely identify a sample. Each bin has a specific mass range with the value of the total intensity of all ions within that range. Bin results are displayed visually as a histogram and can be downloaded in .csv format for further analysis. Binning is a common form of preprocessing for data sets with high dimensionality and is particularly well suited for mass spectrometry data [20,21]. Binning can streamline and simplify the analysis of LCMS-based metabolomic data by reducing its dimensionality [21]. Another advantage of binning is that mass peaks are not tied to retention time indices, and thus represent an unbiased data matrix of all masses in the sample. This allows the user to extract quantitative information from LCMS chromatograms without requiring initial peak deconvolution, a process that is often hindered by poor chromatographic resolution and weak signals. However, chromatographic peaks can be recovered in Haystack as latent variables in EIC plots.

In this paper we demonstrate Haystack's main features and show how it can be used to process LCMS data to identify important features and determine group assignment with comparable accuracy to peak detection methods using the popular XCMS tool.

## Methods

### Technical description

Haystack is a web-based server located at http://binf-app.host.ualr.edu/haystack/. It uses the scripting languages Perl and R and a website interface powered by PHP. Data storage and user information are handled by the MySQL database. Raw LCMS data saved in several common file formats including mzData, mzML, mzXML and netCDF are uploaded directly through Haystack's browser interface. The initial file parsing and TIC and BPC outputs are created by the XCMS R package. The graphical outputs, EIC, and bin analysis are produced by XCMS and MetaboAnalyst R packages [14,16]. A Perl mission control system was implemented to dynamically optimize server resources and performance. All results can be downloaded via comma separated (.csv) files. Sigmaplot 11.0 (Systat Software) was used to generate customized graphs from output files.

### Sample preparation

Tomato plants (*Solanum lycopersicon*) were grown for 6 weeks in a growth chamber under fluorescent lights at either 100 (low light, LL) or 700 (high light, HL) µmol m^-2 ^s^-1 ^photosynthetically active radiation (PAR). The cultivar used was the *h*igh *p*igment-2 dark green (*hp-2*^dg^) mutant strain. For LCMS analysis three independent samples from four plants per treatment were taken for a total of 12 biological replicates for each treatment group. One LL sample was lost during preparation and so the LL dataset consists of 11 samples. Leaf tissue samples (1.2 cm^2^) were frozen in in liquid nitrogen and freeze dried. Samples were extracted in 80% methanol with glass bead homogenization followed by sonication and filtration. Extracts were dried and concentrated 10-fold prior to analysis.

### LCMS analysis

Samples were analyzed by LCMS using an Agilent 1100 HPLC / MSD-VI Ion Trap mass spectrometer with an electrospray ionization (ESI) source. A 250 × 4.6 mm C18 column (Alltech Prevail™) was used with a linear mobile phase gradient consisting of water/0.1% formic acid and methanol. The mass spectrometer was operated in negative ion mode with a dry gas temperature of 350 °C, nebulizer pressure 40 psi, and flow rate 10 liters min^-1^. The trap parameters were set at a scan range of 100-1500 *m/z*; accumulation time 100 ms, target mass 300 *m/z*, compound stability 50%, and trap drive 50%. Mass spectra were collected in profile mode. Chromatogram files were saved in netCDF format from DataAnalysis for LC/MSD Trap Version 5.2 (Agilent Technologies).

### Peak analysis

Peaks were extracted from total ion chromatograms either manually or with the R-based version of XCMS [14]. Manual peak extraction was performed with the Agilent DataAnalysis LC/MSD Trap Software package, v. 5.2. A list of 69 ion peaks common to all samples was obtained by manually scanning total ion chromatograms of representative samples. Extracted ion chromatograms were generated and peak areas were obtained by integration. Both procedures produced a peak table used for subsequent statistical analysis.

### Statistical analysis

Peak data from manual and XCMS peak processing and bin intensity data from Haystack were analyzed using the web-based statistical package MetaboAnalyst 2.0 (http://www.metaboanalyst.ca) [16,22]. Pre-processing steps included filtering, sum normalization, log(2) transformation, and autoscaling. Univariate (fold-change, t-tests, volcano plots) and multivariate (HCA, PCA, PLS-DA) analyses were performed on all data sets.

## Results

Haystack is a web-based server designed to store, display, extract, process, and export LCMS-based metabolomic data. It also can process GCMS data. It has an intuitive browser interface and requires no command line operations, only the ability to use a typical webpage form. Users must register to set up an account. All user files are confidential.

**Figure 1:**
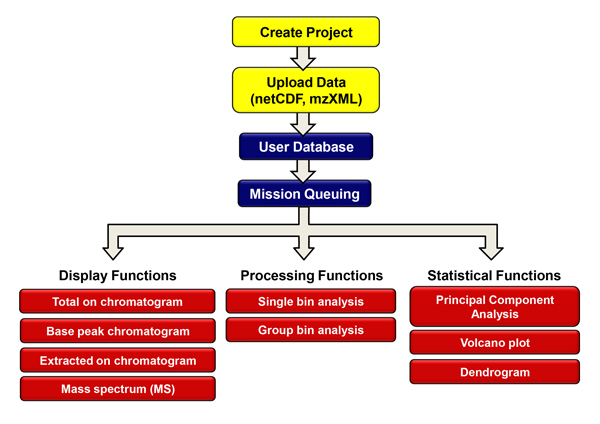
**Workflow of LCMS data processing with Haystack**.

**Figure 2:**
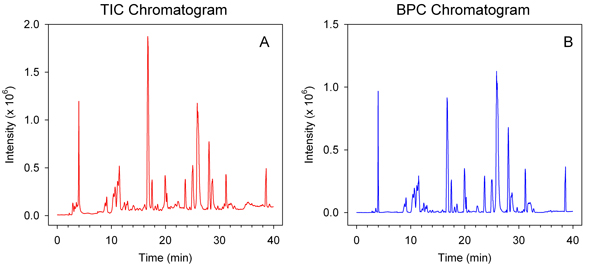
**Representative TIC and BPC plots of LCMS data from a tomato leaf sample**.

Figure 1 shows a summary workflow of data processing steps in Haystack. Raw LCMS data can be saved in several common export formats including netCDF, mzData, mzML, and mzXML. Files are then uploaded into a user database directly through Haystack's browser interface. Files are stored and organized within projects and can be added or deleted as desired. Visualization and processing operations are arranged as tabs within a project. Graphical outputs are displayed as .png files that can be viewed in the browser or saved. Haystack displays the TIC and BPC plots automatically (Figure 2). In many applications involving LCMS data, it is often desirable to display the BPC plot since it contains less baseline noise than the TIC plot. The output of these processes are stored as .csv files within a project.

**Figure 3:**
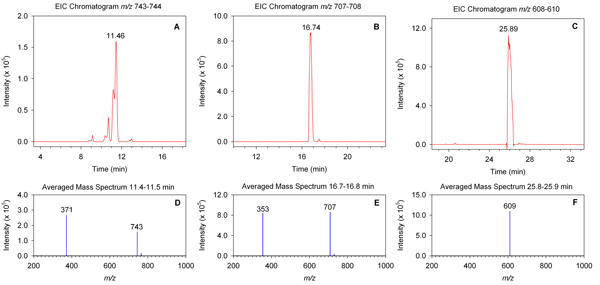
**Representative EICs and mass spectra from a tomato leaf sample**. EICs were generated in Haystack for mass ranges 743-744 (A), 707-708 (B), and 608-610 (C). Mass spectra are shown for time ranges 11.4-11.5 min (D), 16.7-16.8 min (E), and 25.8-25.9 min (F).

Users can generate EICs over any specified mass range by clicking on the 'Generate EIC' tab and entering the starting and ending masses. Users can also generate time-resolved averaged mass spectra by clicking on the 'Generate Mass Spectra' tab and entering the starting and ending times. Haystack displays the EIC and MS plots and provides a .csv export option so data can be imported into a spreadsheet program such as Excel. Figure 3 provides an example of three EICs for the mass ranges 743-744, 707-708, and 608-610 and their corresponding mass spectra. The peaks at 11.46, 16.74, and 25.89 min were subsequently identified as the flavonoids caffeoylglucaric acid, 3-*O*-caffeoylquinic acid (chlorogenic acid), and quercetin-3-*O*-rutinoside (rutin), respectively. The mass specta of caffeoylglucaric acid and chlorogenic acid show the monoisotopic ions (*m/z *371, 353) as well as a dimeric species [2M-1] (*m/z *743, 707) whereas the mass spectrum of rutin shows only the monoisotopic ion (*m/z *609). Users can generate as many EICs and mass spectra as desired, which allows easy comparison of multiple EICs from different samples. If a single file bin analysis is performed, the EICs for all bins are automatically generated and can be downloaded as a zip file, thus providing another data pool that can be mined for peak features. However, it is important to note that a small bin size will result in a large number of EIC files.

**Figure 4:**
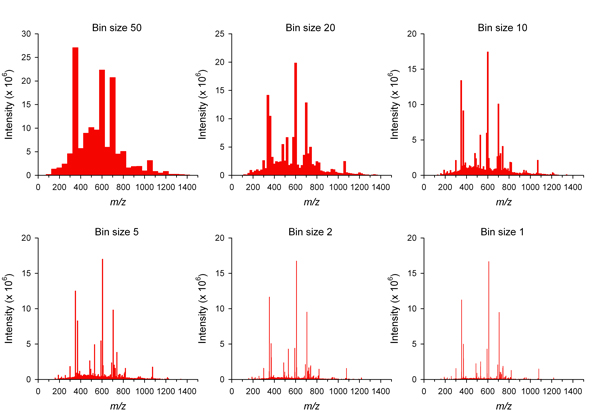
**Bin histograms of LCMS data generated by Haystack for a tomato leaf sample with bin sizes of 50, 20, 10, 5, 2, and 1**.

The bin feature of Haystack parses full-scan LCMS data into uniform bins over any specified mass range. To perform a bin analysis, users simply click on the 'Bin Analysis' tab and enter the bin size. There is also an option to perform a group bin analysis on all files within a project. Haystack returns the total intensity for each bin and displays the results graphically as a histogram. Bin results and can also be downloaded as a .csv file for easy import into a spreadsheet program for display, sorting, and statistical analysis. Figure 4 shows an example of a data set that was analyzed with six different bin sizes (50, 20, 10, 5, 2, 1). The total number of bins (*N*) is given by the formula:

$N=\left(\text{mass range}\right)/\left(\text{bin size}\right)$

It can be seen that there is very little increase in resolution below a bin size of 2. Therefore, we used a bin size of 2 for all subsequent analyses.

Bin results can be used to explore properties of the data, identify important features, and model group assignment. Binning generates a set of interval, non-zero variables in the mass dimension that can be analyzed by univariate (fold change, ANOVA) as well as multivariate (PCA, HCA, PLS-DA) statistical tests. This provides a faster method for processing LCMS data for single or multiple group analysis compared to peak deconvolution methods.

**Figure 5:**
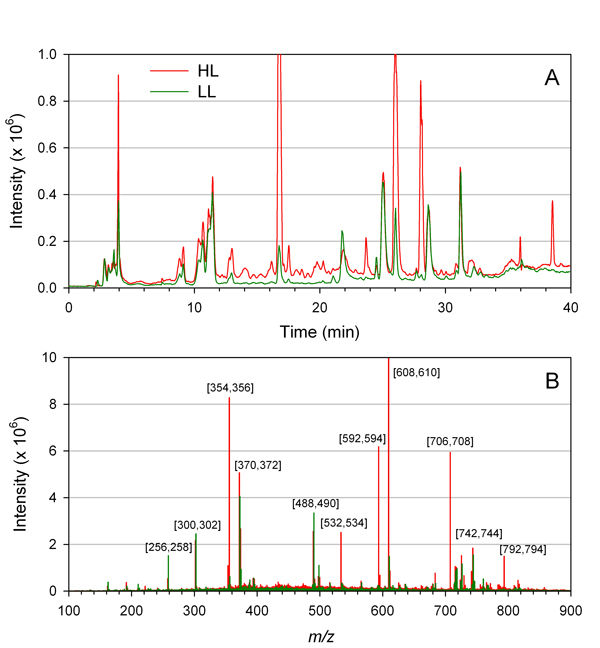
**Comparison of TICs (A) and bin histograms (B) from representative LL and HL samples**. A bin size of 2 was used in this example. High intensity mass bins are indicated.

To demonstrate this idea we analyzed metabolite profiles from tomato plants grown under either high light (HL) or low light (LL) conditions (see Methods for details). Samples were prepared from 12 HL samples and 11 LL samples and investigated by LCMS over a mass range of *m/z *100-1500. Figure 5 shows a representative TIC and bin histogram from each group. Clear differences in the intensity of several peaks can be observed in the chromatograms of the two samples. However, the bin results provide a quantitative "fingerprint" that can be analyzed by chemometric methods to reveal important and often more subtle features. Sorting bin results provides a snapshot of major features. For example, the mass range 608-610 had the highest intensity in the HL sample, whereas the mass range 370-372 had the highest intensity in the LL sample. EICs of these bins identified a peak at 25.8 minutes corresponding to rutin for mass range 608-610 and a series of peaks between 8.8 and 11.5 minutes corresponding to isomers of caffeoylglucaric acid for mass range 370-372 (Figure 3). The masses of other high intensity bins are indicated in Figure 5B.

To test the functionality of Haystack to model class assignment and identify discriminatory features, we used principal component analysis (PCA) to compare the results of bin analysis with manual and automated peak detection methods. For manual peak detection we generated EICs for 69 unique ion peaks in HL and LL samples and integrated the corresponding peaks using Agilent's DataAnalysis Version 5.2 software. For automated peak processing we used the command line version of the open source software XCMS to generate 664 peaks using a peak deconvolution procedure with a default bin size of 0.1. All data sets used in this study are available in Additional file 1.

For each data processing method, we imported the results into MetaboAnalyst 2.0 for statistical analysis [22]. Since, as with many types of chemometric data, binned LCMS data contains a significant amount of baseline noise, removing constant or very weak variables generally improves the analysis [23,24]. Using the interquartile range filter option, MetaboAnalyst removed 25% of the variables (176 out of 700), most of which were at the high and low ends of the mass range and contained mostly baseline signal.

The data were normalized prior to MVA by dividing each variable by the total bin intensity for the sample (sum normalization) to correct for differences in tissue mass between HL and LL samples. The data then were log transformed and autoscaled to improve normality and satisfy equal variance to make features more comparable. The current version of Haystack uses these pre-processing steps as default options for PCA.

**Figure 6:**
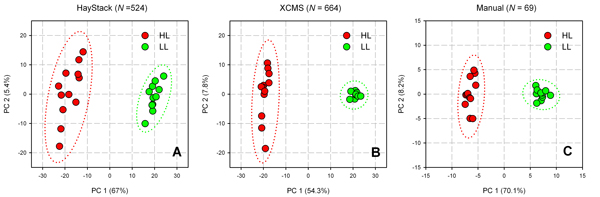
**PCA score plots of LCMS data from LL and HL samples processed using Haystack (A), XCMS (B), or manual extraction (C)**. The number of variables included in the analysis is indicated above each figure.

PCA score plots for the three data processing methods are shown in Figure 6. Each method clearly separated the HL and LL groups along the first PCA axis. There was more scatter observed in the Haystack data as compared with the two peak detection methods, which may be explained by greater variance in binned data since all masses are included in the data set. However, the overall pattern in PCA score plots was similar in all three methods, suggesting that binned data can be used to model group assignment with comparable accuracy to peak detection methods despite a large number of neutral variables.

**Figure 7:**
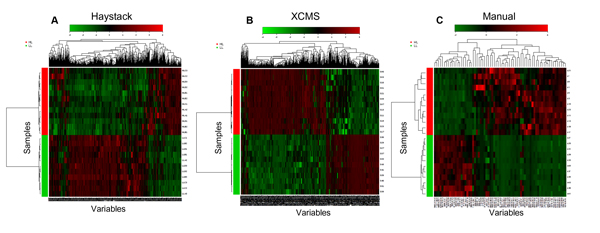
**Hierarchical cluster analysis of data from Haystack (A), XCMS (B), and manual extraction (C) methods**. Pearson's correlation for measure of similarity and Ward's linkage algorithm for clustering were used to generate heatmaps in MetaboAnalyst 2.0.

Multivariate data can also be used to identify discriminatory features. We applied hierarchical cluster analysis (HCA) to each of the three data processing methods to evaluate patterns in the data, and the results were visualized by heatmaps (Figure 7). All three methods produced similar patterns, with HL and LL samples clearly resolved into separate clusters as expected from PCA. Variables were also separated into two major clusters corresponding to the two sample groups. One interesting discrepancy we observed was a greater fraction of variables shown as upregulated in LL samples in the Haystack data set as compared to the two peak detection methods. Although we cannot fully explain this observation, we postulate that the sum normalization pre-processing step may have caused a higher percentage of neutral variables to appear as upregulated in LL samples in the Haystack data set as compared to the other two methods.

**Figure 8:**
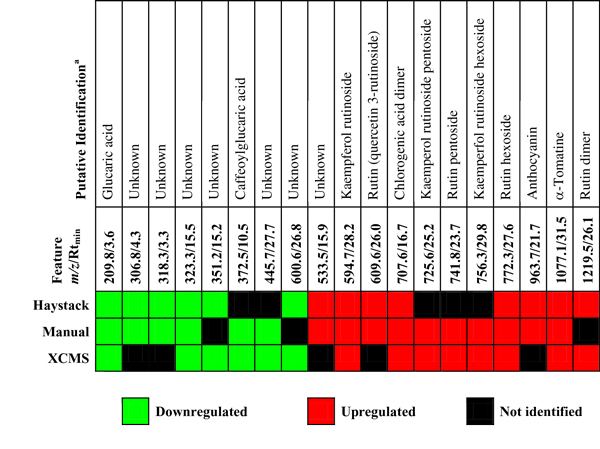
**Comparison of significant features identified by PCA loadings in Haystack, XCMS, and manual extraction**. Features were identified as either downregulated or upregulated in HL samples. Features that were not present in the top 30 PCA loadings are listed as Not Identified.

To further explore the utility of binned data to identify important features, we examined the top 30 PCA loadings in HL and LL groups for each data processing method. We determined retention times from the EICs of significant bins to compare bin results with peak detection results. Peaks were tentatively identified wherever possible based on results of two recent surveys of the tomato fruit metabolome [25,26] as well as MS/MS analysis of selected ions (data not shown). Features were classified as either upregulated or downregulated in HL samples based on normalized mean data. A subset of 19 features were identified as highly discriminatory in at least two of the data processing methods. Figure 8 shows a graphic comparison of the results. The EICs from a representative LL and HL sample for the mass bins in Haystack of each of the corresponding features in Figure 8 is available in Additional file 2.

Although there was good agreement between the three methods, five of the 19 features fell outside of the top 30 PCA loadings in the Haystack analysis. Inspection of the corresponding EICs showed that these bins contained additional peaks to the feature of interest. For example, both manual and XCMS processing methods identified a peak at 25.2 min with *m/z *725.6 that was strongly upregulated in HL samples and was annotated as kaempferol rutinoside pentoside based on published reports [25,26]. The corresponding bin for the mass range 724-726 contained an additional prominent peak at 29.1 min that was present in both LL and HL samples. Consequently, the total bin intensity was not significantly different between the sample groups. Thus, bins containing multiple peaks may not be able to identify discriminatory features. However, both manual and XCMS processing methods also failed to identify several features that were successfully identified by Haystack. We conclude that binning was as effective as peak detection methods in detecting significant features.

**Figure 9:**
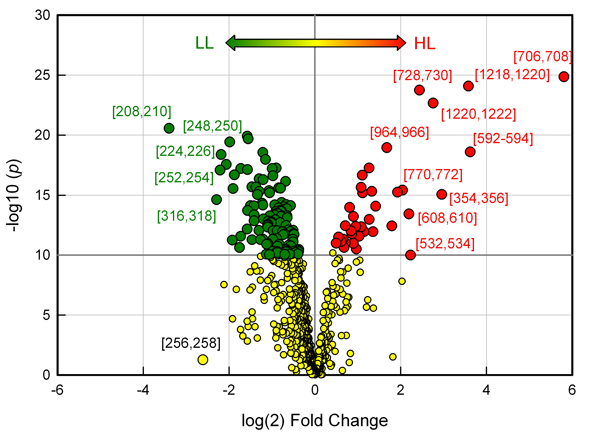
**Volcano plot of bin results comparing HL and LL samples**. Green symbols indicate mass bins that were significantly downregulated while red symbols indicate mass bins that were significantly upregulated in HL samples (*p*<1e^-10^). Significant bins that were upregulated or downregulated by at least 4-fold are indicated.

Volcano plots provide another useful way to identify features that show large differences between groups. Figure 9 shows a volcano plot of bin results from HL and LL samples. Mass bins that show both large magnitude fold-changes and high statistical significance are indicated on the graph. Investigation of the EICs of significant bins were used to identify peaks according to their retention times and mass spectra. We should note that some significant bins may represent the same metabolite feature. For example, mass bins 354-356 and 706-708 both contained a prominent extracted ion peak at 16.7 min that was identified as chlorogenic acid (Figure 3). As stated above, such highly correlated bins are most likely due to common ions in the mass spectra of individual metabolites. Raw bin data cannot distinguish ions that belong to the same metabolite. However, we observed that automated peak detection methods also produced a large number of highly correlated redundant variables.

**Figure 10:**
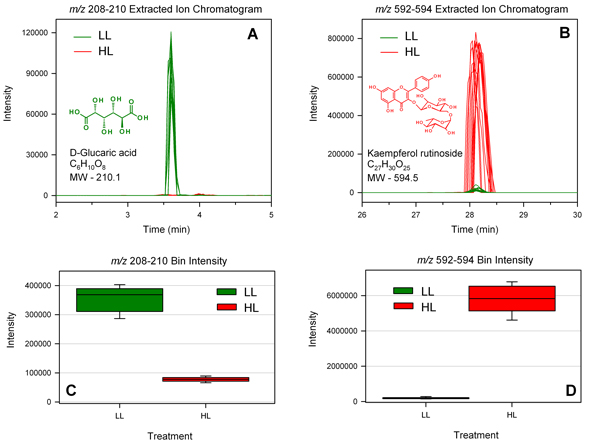
**Example of biomarker identification in LL and HL samples**. Top Panel: EICs of mass bins *m/z *208-210 (A) and *m/z *592-594 (B). Bottom Panel: Box plots of bin intensities for mass bins *m/z *208-210 (C) and *m/z *592-594 (D). The chemical structures and molecular formulae are shown for the annotated peaks corresponding to D-glucaric acid (A) and kaempferol 3-*O*-rutinoside (B).

Figure 10 shows an example of how bin analysis can be used to pinpoint significant features. Mass bin *m/z *208-210 was revealed by volcano plot analysis to be highly represented in LL samples, and this was confirmed by box plot analysis of bin intensity data (Figure 10C). The corresponding overlaid EICs revealed a prominent peak at 3.6 minutes in LL samples which was barely detectable in HL samples (Figure 10A). This peak was identified a D-glucaric acid based on its early elution time and a monoisotopic mass of 209 for the deprotonated ion. D-glucaric acid and its hydroxycinnamate ester caffeoylglucaric acid have been reported in leaves of tomato and *Cestrum euanthes*, a related solanaceous species [27-29]. To our knowledge this is the first report that D-glucaric acid may be a biomarker of LL growth conditions in plants.

A large number of known metabolites were found to be upregulated in HL plants, primarily flavonoids and other phenolic compounds. For example, volcano plot analysis and PCA loadings revealed mass bin *m/z *592-594 to be a highly significant feature of HL samples. The EIC of this bin showed a prominent peak at 28.3 min in HL samples but a very weak signal in LL samples (Figure 10B), and this was confirmed by box plot analysis of bin intensity data (Figure 10D). This peak was subsequently identified as the flavonoid kaempferol 3-*O*-rutinoside based on MS/MS analysis which produced a daughter ion with *m/z *286 corresponding to kaempferol aglycone and a neutral loss fragment of 308 corresponding the to disaccharide rutinose [25,26]. Kaempferol glycosides have previously been observed to be induced by HL in leaves of tomato and bilberry and therefore may represent a biomarker of HL growth conditions in higher plants [30-32].

## Discussion

A significant challenge in untargeted metabolomics is extracting quantitative information from multidimensional LCMS datasets that provides an accurate, comprehensive, and unbiased representation of the metabolome. This is particularly true in plant metabolomics due to the large number of metabolites and their inherent chemical diversity. Plants typically contain hundreds to thousands of unique metabolites that may be present over a large concentration range spanning several orders of magnitude [4,33,34].

In this paper we present Haystack, a new easy to use online platform for metabolomics research. Haystack is a versatile web-based tool that can be used to visualize, parse, and extract information from LCMS datasets rapidly and efficiently. Raw full-scan LCMS data files in one of several common export formats (mzData, mzML, mzXML, NetCDF) can be easily uploaded with a few simple mouse clicks. Users can create projects for multiple file analysis. Haystack's server can store and process a large number of data files, which is often necessary for metabolomics studies.

Haystack automatically generates and displays the total ion chromatogram (TIC) and base peak chromatogram (BPC) when a file is uploaded. Users can search for target masses and obtain extracted ion chromatograms (EICs) with time and intensity values for any mass range. Time-resolved mass spectra can also be generated. A graphical output of all operations is provided, along with a .csv file that the user can export for further analysis or customized graphing. These basic features can be used to extract information from LCMS datasets without requiring technical expertise in command line software tools.

The core function of Haystack is a flexible binning procedure that converts full-scan LCMS data into user-defined mass bins. The resulting histogram provides a graphical representation of the data akin to a "barcode" that uniquely identifies a sample. Sorting by intensity identifies the most abundant masses in a sample. Weak or highly redundant variables can be removed by filtering. When comparing multiple samples, binning creates a set of common interval variables that can be analyzed by chemometric methods such as PCA. A group bin analysis will automatically generate a PCA scores plot, volcano plot, and dendrogram for a two group comparison. For further analysis, group bin results can be imported into a variety of statistical tools. We have found the online tool MetaboAnalyst 2.0 to be particularly useful for analysis of binned mass data since it provides a range of data normalization options as well as a full suite of statistical processing functions [16,22].

The current version of Haystack does not include automated peak detection. However, the EICs of significant bins can be analyzed "by eye" or using signal-to-noise algorithms to identify important peaks. EICs can be generated for any bin or mass range to identify peaks of interest. Unlike traditional MS metabolic fingerprinting methods, which only contain data in the mass dimension, the chromatographic peaks in binned LCMS data are retained as latent variables, so no information is lost during the analysis.

We show that bin analysis can be used as a fast and practical alternative to peak detection methods to streamline and simplify the analysis of LCMS-based metabolomic data. Bin results are less sensitive to weak signals, baseline noise, retention time shifts, and poor chromatographic resolution than traditional peak detection methods, and they do not require complex filtering, smoothing, or alignment pre-processing steps. Mass bins provide an unbiased data matrix of all ions in a sample since mass distributions not tied to specific retention time indices. Unlike peak detection methods, the bin analysis implemented in HayStack has the advantage of differentiating the samples without requiring retention time alignment.

## LCMS data processing methods

Two common approaches for processing LCMS data are manual and automated peak detection [10,24]. Manual methods generally involve identifying peaks in the TIC and producing extracted ion chromatograms (EICs) for the most abundant mass in each peak. Peaks in the EICs are then integrated, usually within the software environment of the instrument, to produce an intensity value for each mass at a given retention time.

An unavoidable drawback with manual peak extraction is that only the most well-defined peaks of relatively abundant metabolites are generally detected, since minor metabolite peaks often cannot be distinguished from baseline noise. This will result in undersampling the data and so significant features may be missed. A second problem arises from the fact that manual extraction within the software environment of most LCMS instruments is time consuming and not feasible for the sample sizes generally required for robust statistical analysis.

The second and more widely used method of LCMS data processing is automated peak detection. This usually involves several pre-processing steps, such as baseline correction, noise reduction, and retention time alignment, followed by peak deconvolution [24]. Automated detection attempts to resolve the TIC into individual component peaks by removing extraneous ion peaks from extracted mass spectra. This approach has been shown to work very well for GCMS data using peak deconvolution tools such as AMDIS combined with data extraction and quantification tools such as MET-IDEA [13]. However, a variety of factors including greater retention time variation, poor chromatographic resolution, isotopic mass spectra, and baseline noise can all create problems with automatic peak detection with LCMS data.

Despite these limitations, a number of software tools are available for processing, visualization, and analysis of LCMS data [12,17,24,35]. A popular and widely used platform for metabolomics data analysis is XCMS [14]. Originally released as a command line tool based in R, XCMS recently has become available in a web-based version known as XCMS Online [18]. Both versions perform automated peak detection on raw full-scan LCMS data using nonlinear retention time alignment, peak detection, and peak matching using a second derivative Gaussian filter. XCMS Online generates and displays EICs, box plots, and mass spectra for all significant features, along with fold-change data and PCA results. An interactive cloud plot feature has been added that offers a range of visualization options. The command line version of XCMS provides a greater range of options for data processing and peak analysis. However, it requires considerably more technical expertise than XCMS Online.

Haystack provides a more flexible data management system than XCMS Online. Single files can be uploaded and processed individually or in zip format for bulk file uploads in Haystack. XCMS Online limits the basic user to a total workspace of 2 Gb, while Haystack has an individual file limit size of 20 Mb but does not limit total number of files for a user. Both Haystack and XCMS Online allow stored datasets to be edited, but HayStacks offers greater flexibility in manipulating stored datasets. Haystack allows the user to create projects in which files can be uploaded. In a project the user can label files with group names and conduct a group bin analysis. There is even a total files list that allows group analysis to be conducted across projects, thus eliminating the need to upload the same files into another group. XCMS Online does not allow files between datasets to be exchanged to form new groups when conducting a new analysis.

In this study we compared metabolite profiles of plants grown under low and high light conditions to generate a data set for differential profiling. Our goal was to test whether Haystack's bin results could accurately predict group assignment and identify discriminatory features in a multivariate analysis. To do this we compared Haystack data with manual and XCMS automated peak processing using the same sample dataset. All three methods gave similar PCA score plots and heatmap patterns, and identified similar important variables by ANOVA, volcano plots and PCA loadings. However, we also observed some notable discrepancies in feature detection between the three methods. Overall, the results appear to validate Haystack's binning function as a means to diagnose both classes and variables in multidimensional LCMS data.

We note that XCMS produced a high percentage (~15%) of missing values in the peak table due to weak or poorly resolved chromatographic peaks. This can be a problem for modeling multivariate data using covariance methods such as PCA [36]. We avoided this problem in manual analysis by deliberately excluding peaks that were not present in at least half the samples of each group. Similarly, all variables in Haystack's bin analysis had a positive value, thus avoiding the missing value problem here as well. Bins that contain mainly baseline noise would be expected to have a weak influence on the covariance matrix, and these were largely removed by filtering during the data pre-processing steps in MetaboAnalyst.

We also observed that XCMS oversampled a large number of peaks in the TIC. Oversampling results in metabolite peaks in the TIC being represented multiple times in the peak table as highly correlated redundant variables. This gives an inflated estimate of the number of unique metabolite peaks in a sample. Oversampling was caused mainly by the default small bin size of 0.1 used in the initial data processing steps in XCMS. Thus, certain aspects of XCMS results should be viewed with caution.

Although binning is one of the critical steps in the automated processing of LCMS data, defining a proper bin size is not a straightforward task [21,37]. Forcing a fixed bin size that is either too large or too small has several disadvantages in peak deconvolution methods. A larger bin size may result in poor resolution of the peaks, while a smaller bin size may split a peak which carries a risk of oversampling the data. In both cases, the outcome is a poorly resolved peak and a less confident integration result. Some authors have proposed variable bin sizes or overlapping bins [14]. One of the problems with applying these methods is the lack of information in the raw data to estimate the parameters for setting these values.

Haystack allows the user to set the bin size. This makes it easy to test different bin sizes to determine an optimal number of variables for a particular dataset, which will depend on the biological complexity of the sample as well as the resolution of the mass spectrometer. Peak deconvolution is not required in Haystack since bin intensity values are not coupled to specific retention times. However, peak information can be recovered by analysis of the time domain of relevant bins. It is inevitable that some bins will contain multiple peaks, since the average number of unique ion peaks increases with bin size.

It should be emphasized that Haystack is not meant to replace conventional peak analysis. An important goal of a metabolomics experiment is to identify all distinct chemical entities in a biological sample, which are best represented by fully resolved and annotated peaks in an LCMS chromatogram. Since binned LCMS data can only provide intensities for specific mass ranges, subsequent data processing is crucial to identify specific ion peaks. Tools such as XCMS can perform global deconvolution of LCMS datasets through peak alignment, filtering, smoothing, etc., and XCMS Online version makes this type of data analysis more accessible for the average user. Table 1 provides a comparison of features in Haystack and XCMS Online. While the latter is an extremely useful tool, we believe that Haystack provides an alternative platform with several unique advantages, particularly the ability to process and visualize raw full-scan LCMS data rapidly and efficiently, and more importantly its unique bin analysis for identifying mass ranges of possible interesting features. Haystack may be considered as the first step in the data analysis pipeline to obtain a snapshot of the data, where XCMS Online may provide a more comprehensive analysis of the data based on peak deconvolution. A version of Haystack that incorporates peak detection is currently under development.

**Table 1:** Comparison of Haystack and XCMS Online features.

Data Visualization	Haystack	XCMS Online
1. Total ion chromatogram (TIC)	Yes	Yes
2. Base peak chromatogram (BPC)	Yes	No
3. User-defined extracted ion chromatograms (EIC)	Yes	No
4. User-defined mass spectra	Yes	No
5. Provide .csv file for all data display outputs	Yes	No
**Data Processing**		

1. User-defined bin analysis of single files or groups	Yes	No
2. Multiple group comparisons	Yes	Yes
3. Retention time correction and peak alignment	No	Yes
4. Significant features based on fold-change	No	Yes
5. Box plots, mass spectra, and EICs of significant features	No	Yes
6. Graphical display of PCA results	Yes	Yes
7. Volcano plot analysis for 2 group comparison	Yes	No
8. Provide .csv file for all data processing outputs	Yes	No

## Conclusions

A major bottleneck in non-targeted metabolomics studies is processing the large amounts of data produced by most metabolomics experiments to obtain useful biological information. In this article we present Haystack, a novel web-based discovery tool that offers a range of data visualization options for LCMS-based metabolomics data and supports non-biased differential profiling studies through a unique and flexible binning function. While other software tools incorporate binning into the initial stages of data processing, only Haystack allows users to export and manipulate raw binned data for statistical analysis. We show that by applying standard chemometric analysis methods, binned data can be used to model class assignment and elucidate significant features without the need for peak deconvolution. The main advantage of this approach is that it is faster and less prone to chromatographic errors than conventional peak extraction methods. Furthermore, average users can upload and process their LCMS data through Haystack's simple web interface without having technical expertise in command line programming languages and software tools. With additional features being engineered such as peak integration and more data processing options, Haystack will become a valuable tool in the workflow of metabolomics research.

## Availability and requirements

• Project name: Haystack

• Project home page: http://binf-app.host.ualr.edu/haystack

• Operating system(s): Platform independent

• Programming language: PHP, R, Perl, MySQL

• Other requirements: Internet browser

• License: free

## List of abbreviation used

ANOVA - Analysis of Variance; BPC - Base Peak Chromatogram; CSV - Comma Separated Value; EIC - Extracted Ion Chromatogram; ESI - Electrospray Ionization; HCA - Hierarchical Cluster Analysis; LCMS - Liquid Chromatography Mass Spectrometry; MS - Mass Spectrometry; MVA - Multivariate Analysis; PCA - Principal Component Analysis; PLS-DA - Partial Least Squared-Discriminant Analysis; TIC - Total Ion Chromatogram.

## Competing interests

The authors declare that they have no competing interests.

## Authors' contributions

SCG conducted the experiments, performed the analysis and drafted the manuscript. SE and HL created the server, designed and implemented the coding for Haystack, participated in the design of the study and helped to draft the manuscript. All authors read and approved the final manuscript.

## Supplementary Material

Additional file 1 **Supplemental information**. Excel file that contains data for 23 samples that were analyzed by the three data processing methods used in this paper. Click here for file

Additional file 2 **Supplemental information**. PDF file that contains EIC plots for the 19 mass bins shown in figure 8 from a representative LL and HL sample. Click here for file

## Declarations

Funding for this publication was provided the National Institutes of Health (P20 GM103429) and the National Science Foundation (EPS-1003970).

This article has been published as part of *BMC Bioinformatics *Volume 15 Supplement 11, 2014: Proceedings of the 11th Annual MCBIOS Conference. The full contents of the supplement are available online at http://www.biomedcentral.com/bmcbioinformatics/supplements/15/S11.
