# Supplementary figures and images for: Haystack, a web-based tool for metabolomics research
Source: BMC Bioinformatics. 2014 Oct 21;15(Suppl 11):S12. doi: 10.1186/1471-2105-15-S11-S12 (PMC4251040; doi:10.1186/1471-2105-15-S11-S12)

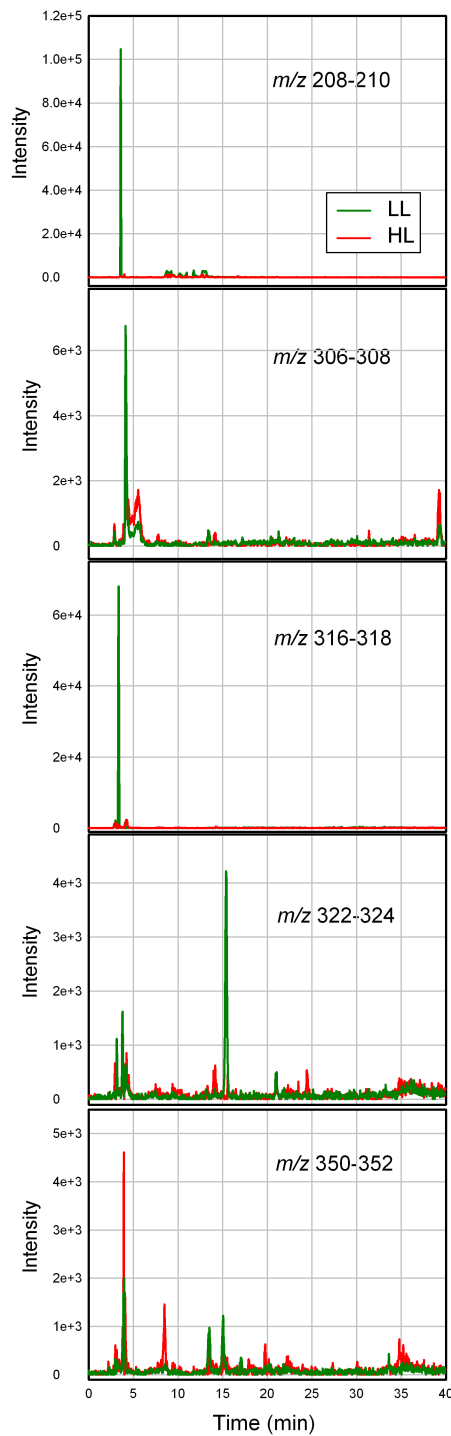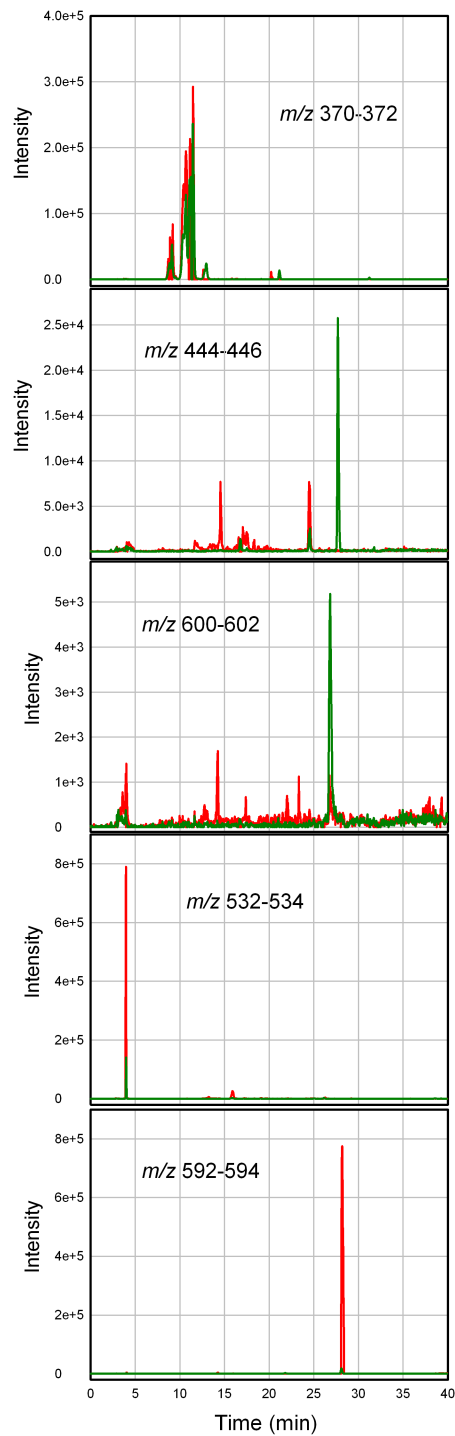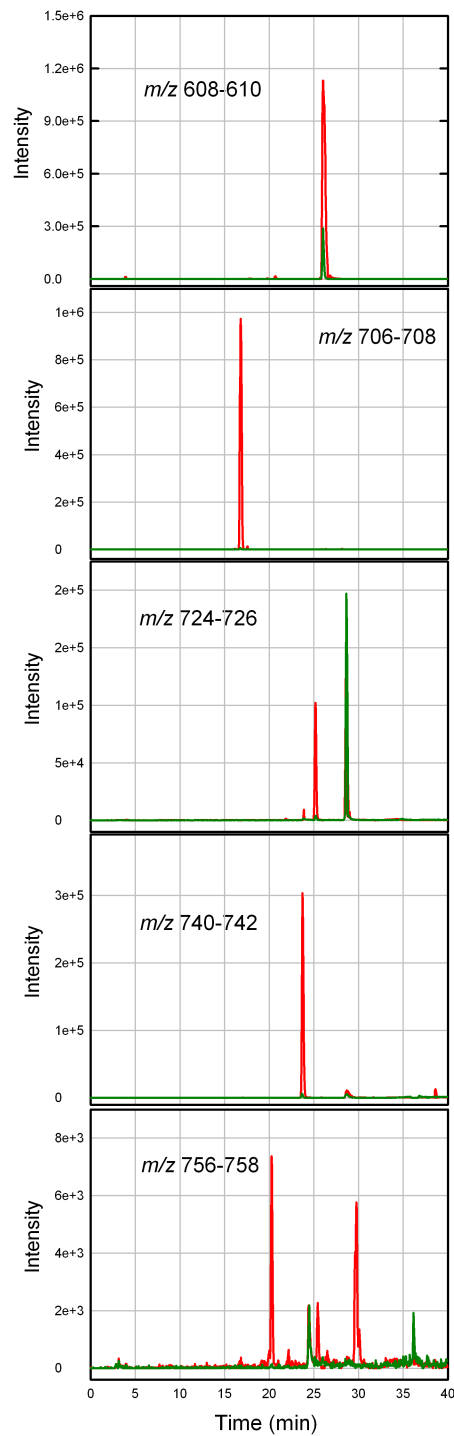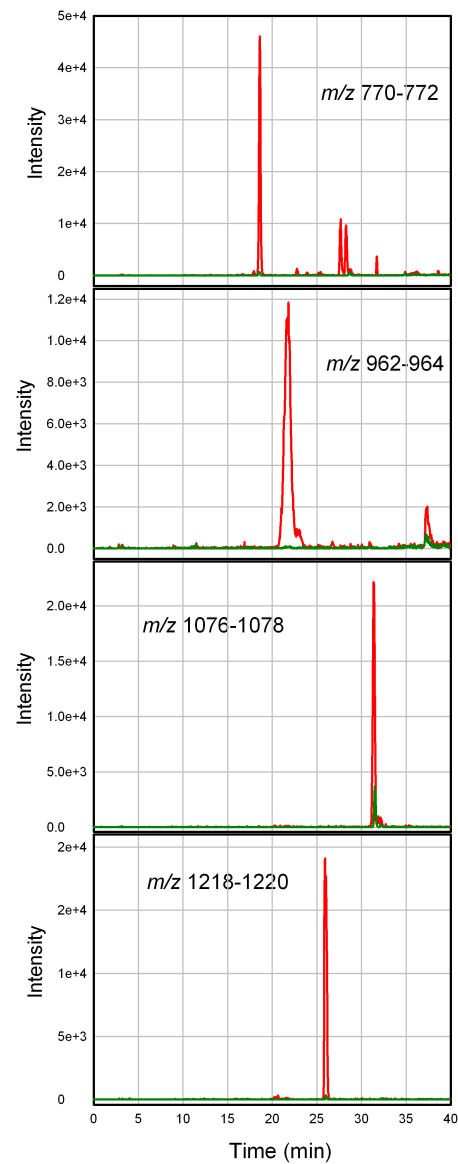

Supplement: Additional file 2 — Supplemental information. PDF file that contains EIC plots for the 19 mass bins shown in figure 8 from a representative LL and HL sample. [file 1471-2105-15-S11-S12-S2.pdf]
